# Maternal Cigarette Smoking and Cleft Lip and Palate: A Systematic Review and Meta-Analysis

**DOI:** 10.1177/10556656211040015

**Published:** 2021-09-27

**Authors:** Matthew Fell, Kyle Dack, Shaheel Chummun, Jonathan Sandy, Yvonne Wren, Sarah Lewis

**Affiliations:** 1 1980 University of Bristol, Bristol, UK; 2 2394 University Hospitals Bristol and Weston NHS Trust, Bristol, UK

**Keywords:** cleft lip and palate, cleft palate, orofacial cleft, pregnancy, smoking

## Abstract

**Objectives:**

A systematic review and meta-analysis to determine the association between active maternal smoking and cleft lip and palate etiology.

**Data sources:**

Medline, Embase, Web of Science and the Cochrane Library from inception to November, 2020.

**Study selection:**

Observational studies of cigarette smoking habits in pregnant women. Outcomes included cleft lip and/or palate, cleft lip  ±  palate and cleft palate only.

**Data analysis:**

Publication bias analyses were performed and the Newcastle Ottawa scales were used to assess study quality. Fixed or random effect models were used in the meta-analysis, dependent on risk of statistical heterogeneity.

**Results:**

Forty-five studies were eligible for inclusion of which 11 were cohort and 34 were case–control studies. Sixteen studies were of sufficient standard for inclusion in the meta-analysis. The summary odds ratio for the association between smoking and cleft lip and/or palate was 1.42 (95%CI 1.27-1.59) with a population attributable fraction of 4% (95%CI 3%-5%). There was limited evidence to show a dose–response effect of smoking.

**Conclusions:**

This review reports a moderate association between maternal smoking and orofacial cleft but the overall quality of the conventional observational studies included was poor. There is a need for high quality and novel research strategies to further define the role of smoking in the etiology of cleft lip and palate.

## Introduction

Cleft lip and/or palate (CL/P) is one of the most common craniofacial birth defects, occurring in approximately 1/700 births (Mossey et al., 2009). It affects children and their families because of appearance and functional difficulties with speech, eating, social interaction, and child development. Seventy percent of children born with CL/P do not have an associated syndrome and the anomaly is believed to be caused by a complex pattern of inheritance with both genetic and environmental influences (Lebby et al., 2010). Defining the role of potentially modifiable environmental factors could reduce the prevalence of this congenital abnormality (Raut et al., 2019). Maternal smoking is a modifiable environmental factor, which is considered a causal factor for CL/P in the 2014 US Surgeon General's Report (United States Department of Health and Human Services, 2014).

Cigarette smoke is a complex aerosol comprising more than 4000 different compounds that can cause harm (Martelli et al., 2015). Maternal smoking has attracted research interest because it is a common exposure and has been established as a risk factor for a spectrum of adverse offspring outcomes including preterm birth, low birth weight, and birth anomalies (Krueger and Rohrich, 2001; Hackshaw et al., 2011). It is biologically plausible that maternal smoking could cause CL/P, although the exact mechanism is unknown (Leite et al., 2002; Krapels et al., 2008). There may be a direct interaction of the smoking products with neonatal tissue, leading to induced hypoxia because of impaired angiogenesis and nicotine-mediated vasoconstriction, which has been shown to disrupt palatal fusion in animal models (Vieira and Dattilo, 2018). An alternative theory is that smoking affects DNA methylation in the fetus, which could impact upon gene expression responsible for lip and palate formation (Lebby et al., 2010).

Three previous meta-analyses have demonstrated weak to moderate links between maternal smoking and CL/P (Wyszynski et al., 1997; Julian Little et al., 2004; Xuan et al., 2016). While previous systematic reviews have been comprehensive, the included studies were not assessed for their quality and this might have compromised the validity of the findings (Crossan and Duane, 2018). Potential sources of bias in the primary studies include no adjustment for confounders, inappropriate control groups and recall bias. There is a need for an updated systematic review with rigorous methodology in this field. We conducted a systematic review and meta-analysis in order to determine the role of active maternal cigarette smoking in the etiology of CL/P.

## Methods

### Identification of Studies

A full protocol of this systematic review, carried out following PRISMA guidance (Moher et al., 2009), was adhered to (see Supplementary Table 1) and is available from the PROSPERO systematic review register (registration number CRD42020222837; https://www.crd.york.ac.uk/prospero/display_record.php?ID=CRD42020222837).

Eligible studies were defined as full-text primary-data publications reporting on pregnant women from the general population who were assessed for prenatal active cigarette smoking. Studies were required to document maternal smoking (either in the peri-conception period or any of the three trimesters) but the assessment of smoking status could have been performed prospectively or retrospectively. Studies of passive (or environmental) maternal smoking or paternal smoking were not included. The protocol included all epidemiological studies using an analytical design whereby an exposed group was compared to an unexposed group. Cohort, case–control, quasiexperimental, natural experiment, family based negative control, and Mendelian randomization study designs were eligible.

The outcome of interest was a live-born child with CL/P or subtypes such as cleft lip only, cleft lip  ±  palate (CL  ±  P), cleft palate only (CP), or submucous cleft. Where studies made a distinction between children born with an isolated cleft or a cleft co-occurring with other anomalies, or where results were provided for those with nonsyndromic and syndromic orofacial clefts separately, effect estimates for isolated and nonsyndromic clefts were extracted preferentially.

Studies were excluded if: full text was unavailable; they were conference proceedings only; they were descriptive studies such as case studies, case series, cross-sectional studies, expert opinion, letter, editorials, or studies using secondary data such as reviews; they were animal studies; or there was insufficient data to estimate the effect size of the association between maternal smoking and CL/P (see Supplementary Table 2 for exclusion and exclusion criteria).

The databases searched included Medline, Embase, the Web of Science, and the Cochrane Library from inception to November 9, 2020. The search was tailored individually to each database with input from a University Librarian (see Supplementary Figures 1 to 4 for search strategies) and there was no language restriction. The search focused on published literature and did not include gray literature. In addition, manual searches of reference lists of recent relevant systematic reviews and all studies included in the systematic review were performed.

Titles and abstracts were reviewed independently by two reviewers (MF/KD) according to the specified inclusion/exclusion criteria and differences resolved through discussion to reach a consensus. Where an abstract was not available or where a decision on inclusion/exclusion could not be reached by reviewing the abstract alone, full-text screening was similarly performed independently by two reviewers for inclusion and any disagreements resolved through discussion. When multiple reports of a study were identified, the study with the greatest number of patients was selected. The Rayyan web application was used to facilitate the screening process (Ouzzani et al., 2016).

### Data Extraction

Data was extracted via Microsoft Forms into an excel spreadsheet. Data extracted included: title, authors, publication year, country of the study population, study design, sample description, sample size, outcomes recorded, confounding factors measured, and study outcomes including dose–response data. Adjusted measures of effect were extracted preferentially to reduce the impact of confounding factors.

### Assessment of Study Quality

The Newcastle Ottawa Scale (NOS) (Wells et al., 2021) was used to assess the quality of cohort and case–control studies included in this systematic review. The NOS for cohort studies consists of eight questions among three domains (selection, comparability, and outcome). Similarly, the NOS for case–control studies consists of eight questions among three domains (selection, comparability, and exposure). Stars are awarded for adequate methodology and were used to allocate a score of good, fair, or poor to each study with predefined criteria (see Supplementary Table 3). Good and fair studies were deemed appropriate for meta-analysis, whereas studies categorized as poor were deemed to be of too low quality for inclusion.

From multiple potential confounding factors that are thought to influence cleft etiology, we selected four factors that were supported by the strongest evidence base to enable assessment in the NOS comparability domain (Wells et al., 2000). There is strong prior evidence that maternal age can influence chromosomal anomalies and that maternal alcohol consumption can influence facial development (Bille et al., 2005; Molina-Solana et al., 2013). Studies were required at least to adjust for maternal age and alcohol consumption in order to achieve a “fair” rating and be included in the meta-analysis. Weaker evidence from observational studies suggest folic acid supplementation and obesity may be risk factors for OFC (Badovinac et al., 2007; Izedonmwen et al., 2015). The adjustment for additional confounding factors was reported but did not form a part of the NOS quality assessment. Data extraction and assessment of study quality was performed by one reviewer (MF) and checked for accuracy by a second reviewer (KD) (Centre for Reviews and Dissemination, 2009).

Funnel plots were used to visually assess the likelihood of small study publication bias if more than 10 studies were included, although asymmetry in the funnel plot can also be due to true heterogeneity of the treatment effect, sampling variation, and poor study design (Sterne et al., 2011). Egger's test was calculated to quantify funnel plot asymmetry.

### Data Synthesis

A descriptive summary and narrative analysis of the included studies was performed, alongside an indication of study quality, in accordance with published guidance (Popay et al., 2006). The heterogeneity of the included studies was analyzed by exploring the study characteristics and using the *I*^2^ statistic where sufficiently similar studies were meta-analyzed.

The quantitative impact of the association between maternal smoking and orofacial clefting was investigated using meta-analysis techniques where studies met the quality criteria for inclusion and shared sufficient methodological homogeneity. The minimum number of studies to conduct a meta-analysis was two. Pooled estimates for binary outcomes were calculated using the inverse variance method. The odds ration (OR) was the principle summary measure extracted from the primary studies and meta-analyzed. The fixed-effects model was used where levels of statistical heterogeneity were low (*I*^2^ < 50%); otherwise the random-effects model was used. The population attributable fraction (PAF) was calculated to assess the public health impact (Mansournia and Altman, 2018) using the pooled odds ratio and the prevalence of exposure among cases (Miettinen, 1974). The dose–response impact of maternal smoking was analyzed for studies in which the smoking dose categories used by the included studies were analogous. Subgroup meta-analysis of the smoking dose categories was performed using the random-effects model. Meta-analysis was performed using the “meta” package (Harrer et al., 2021) via the R Project for Statistical Computing (http://www.R-project.org/).

## Results

### Study Selection and Study Characteristics

**Figure 1.**
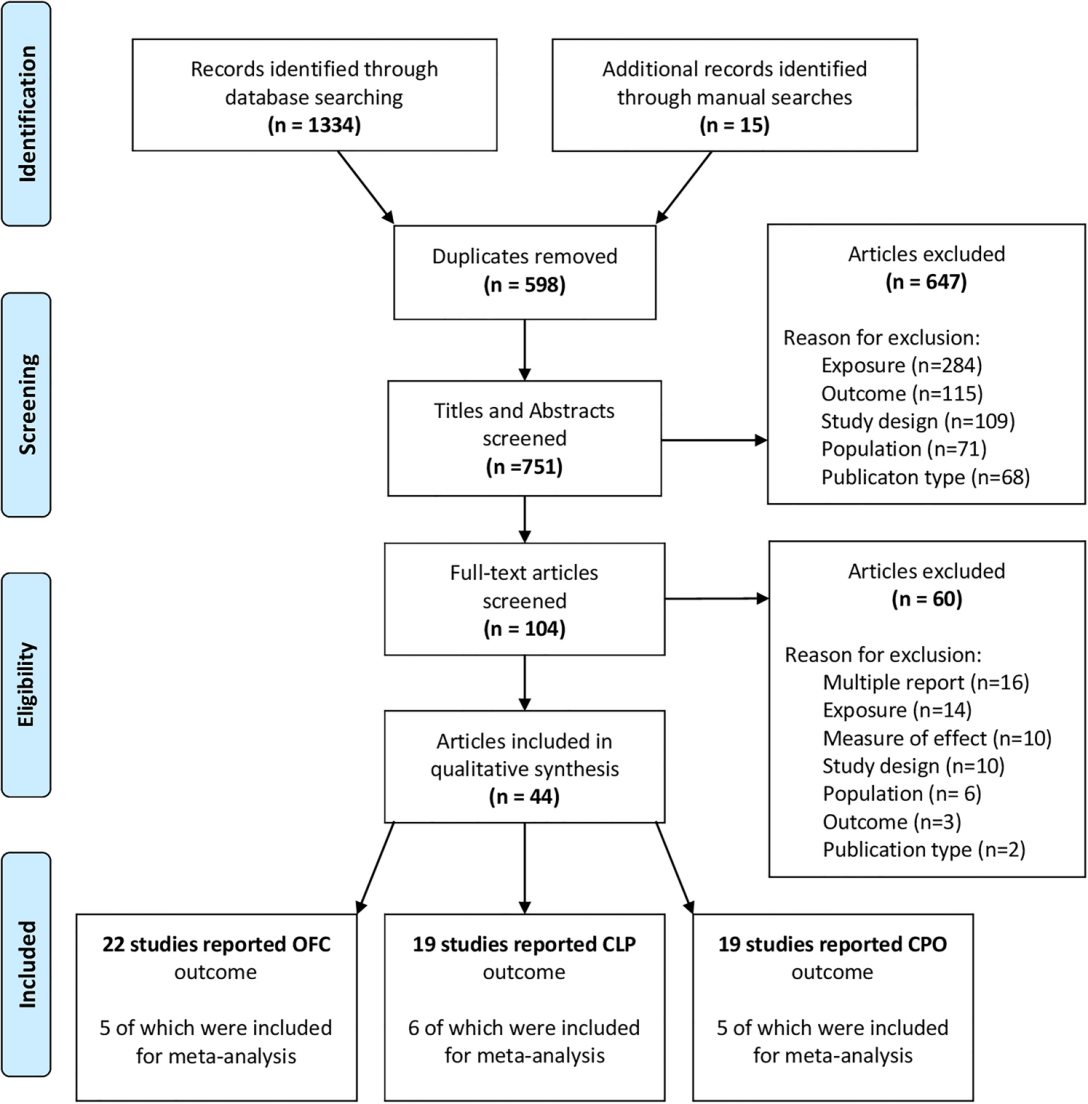
A flow chart of the search strategy and study selection.

A flowchart for the article review process is shown in Figure 1. A total of 1334 citation records were identified from searching the four databases. A manual search of relevant systematic reviews and included studies identified 15 additional studies. After exclusions (see Supplementary Table 4), 45 studies from 44 publications were included in the systematic review (one publication reported two case–control studies from distinctly separate populations (Shi et al., 2007); 11 cohort studies and 34 case–control studies (see Table 1). The earliest study to be included in the review was published in 1986 (Shiono et al., 1986). In total, 28 405 mothers giving birth to a live-born child with CL/P have had their smoking status during pregnancy analyzed among the 45 studies.

**Table 1.** Characteristics of Included Studies.

Author	Year	Country	Period	Sample details	Control details	No. of cases (proportion exposed)	No. of controls (proportion exposed)	Period of smoking	Effect of smoking dose	Outcome
Cohort studies										
Shiono et al.	1986	USA	1974-1977	13 Northern California Kaiser Clinics	Births with no congenital deformity	56 (27%)	NS	T1	No	CL ± P and CP
Malloy et al.	1989	USA	1980-1983	Missouri Centre for Health Statistics Multisource Birth Defects Registry	NS	451 (NS)	288067 (NS)	T1-3	No	CL/P
McDonald et al.	1992	USA	1982-1984	Survey in Montreal	Births matched to location and date	96 (39%)	89317 (33%)	T1	Yes	CL/P
Kallen	1997	Sweden	1983-1992	The Swedish Registry of Congenital Malformations and the Medical Birth Registry	Births with noncleft congenital deformities	1634 (31%)	1002742 (27%)	T1	No	CL ± P and CP
Woods et al.	2001	USA	1998-1999	The TriHealth Hospitals in Cincinnati	Births with and without noncleft congenital birth defects	7 (14%)	18076 (11%)	T1-3	No	CL/P
DeRoo et al.	2003	USA	1987-1990	Washington State Birth Defects Registry (BDR)	Birth with noncleft congenital deformities matched to location and date	608 (23%)	297 530 (21%)	T1	No	CL ± P and CP
Bille et al.	2007	Denmark	1997-2003	The Danish National Birth Cohort	Noncleft births	192 (32%)	880 (25%)	T1	Yes	CL/P, CL ± P and CP
Lebby et al.	2010	USA	2005	US Natality Database	Births without a congenital deformity	1654 (18%)	1654 (10%)	T1-3	No	CL/P
Gunnerbeck et al.	2014	Sweden	1999-2009	Swedish Medical Birth Register	Noncleft births	1985 (10%)	1086213 (8%)	P	No	CL/P
Leite et al.	2014	Denmark	1997-2010	Danish Medical Birth Register	Noncleft births	1564 (23%)	838265 (19%)	T1	No	CL ± P and CP
Sato et al.	2020	Japan	2011-2014	Japan Environment and Children's Study	Noncleft births	146 (16%)	94 174 (13%)	T1	No	CL ± P
Case-control studies										
Khoury et al.	1989	USA	1968-1980	Atlanta Birth Defects Case–Control Study	Births matched to location and date	345 [41%]	2809 [NS]	P	Yes	CL ± P and CP
Van Den Eeden et al.	1990	USA	1984-1986	Washington State Birth Records	Births without a congenital malformation matched to date	173 [NS]	4500 [23%]	T1-3	NS	CL ± P and CP
Hwang et al.	1995	USA	1984-1992	Maryland Birth Defects Reporting and Information System [BDRIS]	Births with non-cleft congenital deformities	183 [37%]	284 [29%]	T1-3	No	CL ± P and CP
Shaw et al.	1996	USA	1987-1989	California Birth Defects Monitoring Programme	Births matched to location and date	731 [32%]	734 [23%]	P	Yes	CLP and CPO
Lieff et al.	1999	USA	1976-1992	Slone Epidemiology Unit Birth Defects Study	Births with noncleft congenital deformities	1072 [36%]	2295 [30%]	T1-3	Yes	CL, CL ± P and CP
Lorente et al.	2000	France/UK/Italy and Netherlands	1989-1992	European Registration of Congenital Anomalies	Consecutive births or births matched to location and date	133 [37%]	1134 [NS]	T1	Yes	CL ± P and CP
Chung et al.	2000	USA	1996	US Natality Database	Births without a congenital malformation	2207 [21%]	4414 [15%]	T1-3	Yes	CL/P
Beaty et al.	2001	USA	1992-1998	The Maryland Birth Defects Reporting and Information System [BDRIS] and the Children's National Medical Centre in Washington DC	Births without a congenital deformity identified from clinical settings	135 [20%]	152 [14%]	P	No	CL ± P and CP
Wyszynski and Wu	2002	USA	1997	US Natality Database	Births without congenital deformities	2029 [19%]	4050 [17%]	T1-3	Yes	CL/P
Little et al.	2004	UK	1997-2000	UK Cleft Teams	Non-cleft births	190 [42%]	248 [24%]	T1	Yes	CL ± P and CP
Meyer et al.	2004	Sweden	1983-1997	Swedish Medical Birth Registry	Non-cleft births	1853 [30%]	128 688 [24%]	T1	YEs	CL ± P and CP
Krapels et al.	2006	Netherlands	1998-2003	Netherlands Cleft Teams	Births without a congenital malformation	350 [25%]	222 [23%]	P	Yes	CL ± P and CP
Shi et al.(A)	2007	Denmark	1991-1994 [DBS]	Danish Case–Control study [DBS]	Noncleft birth recruited from same hospital as case mother	270 [40%]	485 [32%]	P	Yes	CL ± P and CP
Shi et al.(B)	2007	USA	1987-2001	Iowa Registry for Congenital and Inherited Disorders	Births without congenital deformities matched by sex and date	379 [27%]	397 [20%]	P	Yes	CL/P, CL ± P and CP
Grewal et al.	2008	USA	1999-2003	Hospital reports in California	Births without congenital deformity recruited from same hospital as case mother	701 [9%]	700 [18%]	P	Yes	CL ± P and CP
Lie et al.	2008	Norway	1996-2001	Norway Cleft Teams	Noncleft births	573 [42%]	763 [32%]	T1	Yes	CL ± P and CP
Chevrier et al.	2008	France	1998-2001	7 French Hospitals	Births without congenital deformity recruited from same hospital as case mother	240 [28%]	236 [29%]	T1	Yes	CL ± P and CP
Leite and Koifman	2009	Brazil	Not stated	Nossa Senhora de Loreto Municipal Hospital, Brazil	Births without congenital deformity recruited from same hospital as case mother	274 [19%]	548 [16%]	T1	Yes	CL/P
Mirilas et al.	2011	Greece	2004-2009	Single Greek Hospital	Noncleft children presenting to the hospital surgical department	35 [17%]	35 [20%]	T1	No	CL/P
Zhang et al.	2011	China	2006-2009	University of Harbin Medical University, China	Births without congenital deformity recruited from same hospital as case mother	304 [5%]	453 [1%]	P + T1	Yes	CL, CL ± P and CP
Ibarra-Lopez et al.	2013	Mexico	not stated	2 hospitals in Mexico	Noncleft children presenting to the involved hospitals	88 [1%]	116 [7%]	T1	No	CL/P
Salihu et al.	2014	Kosovo	1996-2005	NS	NS	244 [NS]	488 [NS]	T1-3	No	CL/P
Bezerra et al.	2015	Brazil		2 hospitals in Brazil	Noncleft children recruited from schools	140 [14%]	175 [13%]	T1		CL/P
Hao et al.	2015	China	2009-2014	3 hospital sites in China	Births without congenital deformity recruited from same hospital as case mother	499 [7%]	480 [6%]	T1-3	No	CL ± P and CP
Martelli et al.	2015	Brazil	2009-2012	Single hospital in Brazil	Births without congenital deformity recruited from same hospital as case mother	843 [25%]	676 [14%]	T1	No	CL/P, CL ± P and CP
Figueiredo et al.	2015	DRC, Vietnam, Philippines and Honduras	2009-2014	Operation Smile International Missions	Births without congenital deformity recruited from same hospital as case mother	430 [1%]	754 [<1%]	T1	No	CL/P
Ebadifar et al.	2016	Iran	2013-2015	Single center in Iran	Noncleft children from Iran	105 [39%]	218 [2%]	T1	No	CL/P
Liu et al.	2016	China	2002-2014	Shanxi Province, China	NS	205 [<1%]	1223 [2%]	P	No	CL/P
Angulo-Castro et al.	2017	Mexico	2010-2015	Single hospital in Mexico	Noncleft births recruited from same hospital as case mother	24 [46%]	24 [13%]	T1-3	No	CL/P
Xu et al.	2018	China	2013-2016	Single hospital in China	Children with frenulum abnormality recruited from same hospital	236 [21%]	209 [6%]	T1-3	No	CL/P
Raut et al.	2019	USA	1997-2011	National Birth Defects Prevention Study	Births without congenital deformities	4003 [23%]	11 395 [18%]	P	No	CL ± P and CP
Acs et al.	2020	Hungary	1980-2009	Hungarian Congenital Abnormality Registry	Births without congenital deformities	751 [19%]	1196 [8%]	T1	No	CP
Regina et al.	2020	Brazil	2012-2014	Cleft unit at Brazilian Hospital	Births without congenital deformities	150 [9%]	300 [5%]	T1-3	No	CL/P
Auslander et al.	2020	Vietnam, Philippines, Honduras, Nicaragua, Morocco, Congo and Madagascar	2012-2017	Operation Smile Internatinal Missions	Births without congenital deformities recruited from surrounding regions	2137 [<1%]	2014 [<1%]	T1-3	No	CL/P and CL ± P

Abbreviations: CL, cleft lip only; CL/P, cleft lip and/or cleft palate; CL  ±  P, cleft lip  ± palate; CP, cleft palate only; NS, not stated; P, peri-conceptual; T1, first trimester; T1-3, anytime during pregnancy.

### Reported Outcomes

Twenty-two studies reported on CL/P outcome, 19 studies reported on CL  ±  P outcome, and 19 studies reported on CP outcome. The effect estimates from these studies reporting on each of the three outcomes appeared to be symmetrically distributed according to the funnel plot and Egger's test, indicating that publication bias is unlikely to have influenced our findings (see Supplementary Figure 5). As only two studies reported with cleft lip alone as the outcome, a funnel plot was not performed for these. Nine studies reported smoking dose–response effects for CL/P outcome, a further 14 studies gave results by smoking dose for CL  ±  P as the outcome and 13 studies for CP as the outcome.

Table 2 shows the study quality assessment for cohort and case–control studies based on the NOS. Only one study (Raut et al., 2019) of the 45 included studies had low scores in all eight NOS questions. Three studies were deemed to be good quality, 13 studies were deemed fair quality, and 29 deemed poor quality and the latter were excluded from the meta-analysis. A greater proportion of cohort studies (5 of 11) met the quality threshold for meta-analysis inclusion than case–control studies (11 of 34). The most common area lacking was the failure to adjust for confounding factors. The potential for exposure recall bias was present in all 34 of the case–control studies as by definition, information on exposure was collected retrospectively. Only four out of 11 cohort studies collected maternal smoking exposure data prospectively.

**Table 2.** Quality Assessment of Included Studies Using the Newcastle Ottawa Scale.

Author Cohort studies	Year	Selection	Comparability	Outcome	Quality score
Shiono et al.	1986	★★★	★	★★★	Fair
Malloy et al.	1989	★★		★★	Poor
McDonald et al.	1992	★★★	★	★★	Fair
Kallen	1997	★★★★		★★★	Poor
Woods et al.	2001	★★★★		★★	Poor
DeRoo et al.	2003	★★		★★★	Poor
Bille et al.	2007	★★★★	★	★★★	Fair
Lebby et al.	2010	★★★	★	★★	Fair
Gunnerbeck et al.	2014	★★★★		★★★	Poor
Leite et al.	2014	★★★★		★★★	Poor
Sato et al.	2020	★★★	★★	★★★	Good
Case–Control Studies		Selection	Comparability	Exposure	
Khoury et al.	1989	★★★★	★	★	Poor
Van Den Eeden et al.	1990	★★★	★	★★	Fair
Hwang et al.	1995	★		★	Poor
Shaw et al.	1996	★★★★	★	★★★	Fair
Lieff et al.	1999	★★		★★	Poor
Lorente et al.	2000	★	★	★	Poor
Chung et al.	2000	★★★	★	★★	Fair
Beaty et al.	2001	★★	★	★	Poor
Wyszynski and Wu	2002	★★★	★	★★	Fair
Little et al.	2004	★★		★★	Poor
Meyer et al.	2004	★★★		★★★	Poor
Krapels et al.	2006	★★		★	Poor
Shi et al.(A)	2007	★		★★	Poor
Shi et al.(B)	2007	★★★★	★	★	Poor
Grewal et al.	2008	★★★★	★★	★★	Good
Lie et al.	2008	★★★★	★	★★	Fair
Chevrier et al.	2008	★★	★	★★	Fair
Leite and Koifman	2009	★★	★	★	Poor
Mirilas et al.	2011	★★		★	Poor
Zhang et al.	2011	★★	★	★	Poor
Ibarra-Lopez et al.	2013	★★		★	Poor
Salihu et al.	2014				Poor
Bezerra et al.	2015	★★★		★	Poor
Hao et al.	2015	★	★	★	Poor
Martelli et al.	2015	★★		★	Poor
Figueiredo et al.	2015	★★		★	Poor
Ebadifar et al.	2016	★★		★	Poor
Liu et al.	2016	★		★★	Poor
Angulo-Castro et al.	2017	★		★	Poor
Xu et al.	2018	★★	★	★★	Fair
Raut et al.	2019	★★★★	★★	★★★	Good
Acs et al.	2020	★★★★	★	★★	Fair
Regina et al.	2020	★★		★	Poor
Auslander et al.	2020	★★	★★	★★	Fair

Good quality: 3 or 4 stars (★) in selection domain AND 2 stars in comparability domain AND 2 or 3 stars in outcome/exposure domain; Fair quality: 2 stars in selection domain AND 1 or 2 stars in comparability domain AND 2 or 3 stars in outcome/exposure domain; Poor quality: 0 or 1 star in selection domain OR 0 stars in comparability domain OR 0 or 1 stars in outcome/exposure domain.

All of the 11 cohort studies were truly or somewhat representative of the general population and were able to demonstrate the outcome of interest was not present at the start of the study. Of the case–control studies, 7 out of 34 did not meet the participant selection domain criteria due to failing to demonstrate independent validation of case definition (11 of 34), the potential for selection bias of cases (23 of 34), and/or selected controls from hospitalized populations (21 of 34).

Comparability criteria was not met in 6 out of 11 cohort studies and 20 out of 34 case–control studies due to not adjusting for at least maternal age and maternal alcohol consumption as confounders in the analysis. Folic acid supplementation and obesity were adjusted for in less than half of included studies (see Supplementary Table 5).

All of the 11 cohort studies used record linkage to verify OFC outcome. Exposure criteria were not met by 18 out of 34 case–control studies because of relying on self-assessment (8 of 34), using an interviewer who was not blinded to case/control status (23 of 34), and/or the non-response rate of cases/controls was not described (20 of 34).

### Meta-Analysis

**Figure 2.**
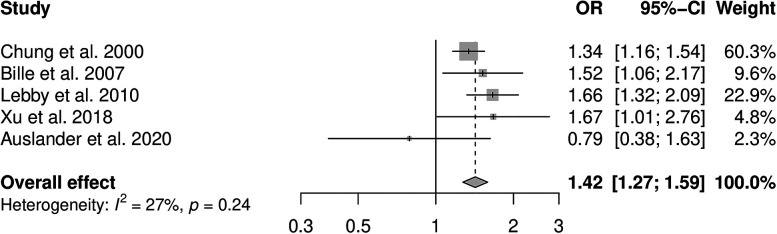
Forest plot to display the measures of effect for studies reporting cleft lip and/or palate outcome. The overall effect has been calculated using a fixed-effects method.

Five studies reporting effect estimates for smoking and CL/P were included in the meta-analysis (see Figure 2). There was no strong evidence of between study heterogeneity (*I*^2^ = 27%, *P* = .24). The pooled OR using the fixed-effects model was 1.42 (95% CI: 1.27, 1.59). Based on the proportion of maternal smoking among case mothers of 14% in these five studies, the PAF was 4% (95% CI: 3%, 5%).

**Figure 3.**
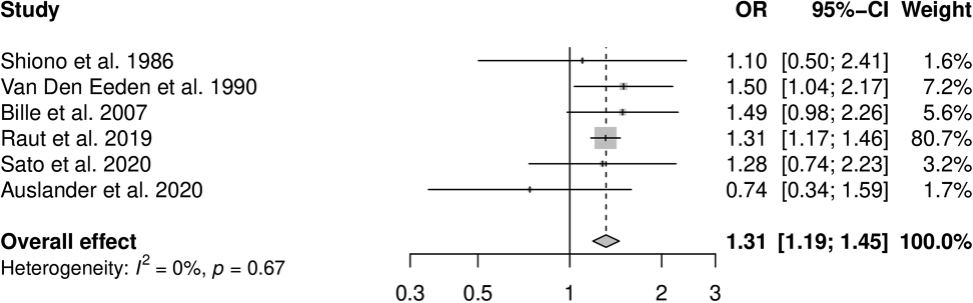
Forest plot to display the measures of effect for studies reporting cleft lip  ±  palate outcome. The overall effect has been calculated using a fixed-effects method.

**Figure 4.**
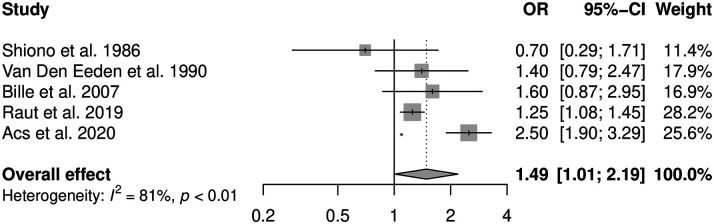
Forest plot to display the measures of effect for studies reporting cleft palate only outcome. The overall effect has been calculated using a random-effects method.

Six studies reporting the effect for smoking and CL  ±  P were included in the meta-analysis (see Figure 3). There was no evidence for statistical heterogeneity between the studies (*I*^2^  =  0%, *P* = .67). The pooled OR using the fixed-effects model was 1.31 (95% CI: 1.19, 1.45). Five studies reporting measures of effect for smoking and CP were included in the meta-analysis (see Figure 4). The statistical heterogeneity between the studies was high (*I*^2^  =  81%, *P* < .01) due to an outlying case–control study performed in Hungary (Ács et al., 2020), reporting a stronger positive effect of smoking on CP than the other included studies. The pooled OR using the random-effects model was 1.49 (95% CI: 1.01, 12.19). The exclusion of the outlying study in the CP meta-analysis resulted in no evidence for statistical heterogeneity (*I*^2^  =  0%, *P* = .49) and a fixed effect pooled OR of 1.25 (95% CI: 1.09, 1.44). It was not possible to calculate the PAF for maternal smoking and CL  ±  P or CP due to missing data in included studies, precluding calculation of the prevalence of exposure.

Individual study effect estimates and pooled analysis for all studies included in this systematic review reporting outcomes for CL/P, CL  ±  P, and CP can be found in Supplementary Figures 6 to 8.

### Subgroup Analysis

**Figure 5.**
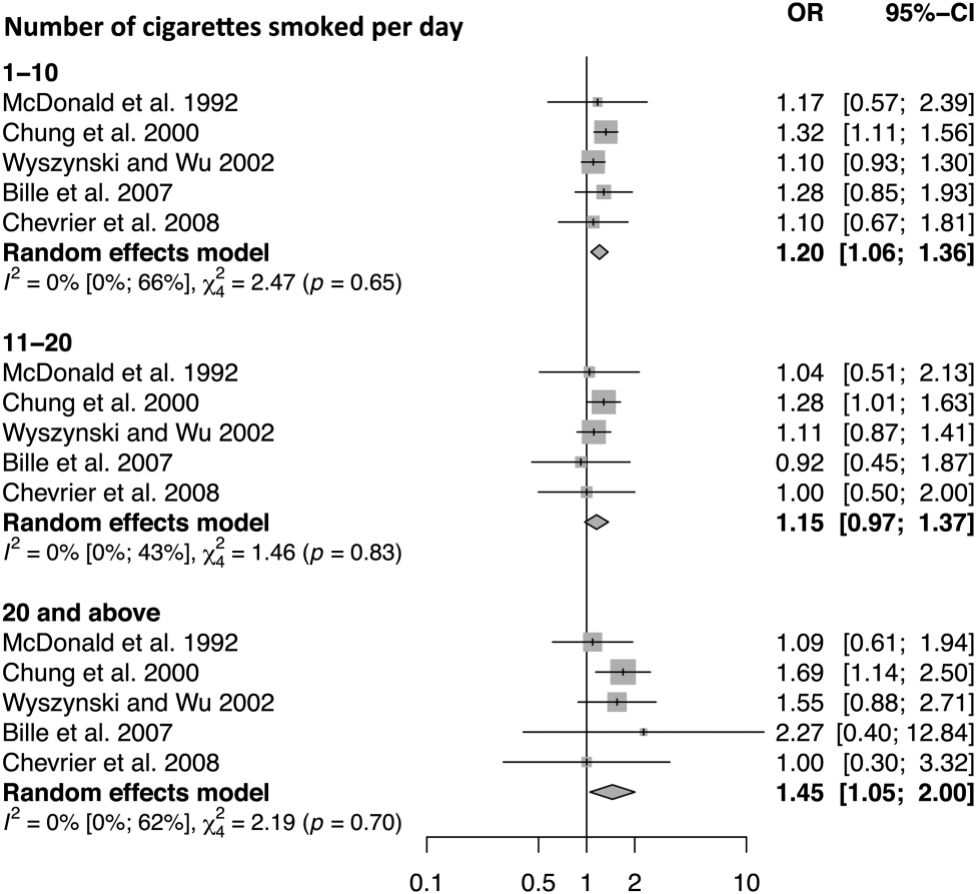
A subgroup forest plot to display the dose–response effect of smoking on cleft lip and/or palate outcome. The overall effect for each of the three-dose categories (1-10, 11-20, and >20 cigarettes smoked per day) has been calculated using a random-effects model.

Five studies reporting measures of effect for the dose of smoking and CL/P were included in the subgroup meta-analysis (see Figure 5). All five studies measured three doses of smoking (low, medium, and high) with comparable numbers of cigarettes smoked per day at each dose (1-10, 1120, and >20 cigarettes per day). The pooled OR for the lowest dose of smoking was 1.20 (95% CI: 1.06, 1.36), for intermediate dose was 1.15 (95% CI: 0.97, 1.37) and the highest dose was 1.45 (95% CI: 1.05, 2.00).

Four studies were eligible for inclusion into the meta-analysis of the effect of smoking dose for both CL  ±  P and CP, respectively, but it was not possible to perform a meta-analysis because the reported smoking dose levels were not comparable.

## Discussion

### Summary of Evidence

There has been a large body of work to investigate the role of active maternal smoking in CL/P etiology, as shown by the 45 studies that met our inclusion criteria. This high volume of research should have provided a clear indication of the association between maternal smoking and CL/P, but the poor quality of studies overall has compromised the validity of the reported findings. Only three studies out of the 45 included in this review were judged to be of good quality (Grewal et al., 2008; Raut et al., 2019; Sato et al., 2020). The most common reason for poor quality within the studies was a failure to adjust for recognized confounding factors, placing the analyses at high risk of bias. Mother's age, alcohol intake and obesity are all strongly associated with smoking behavior and all have been hypothesized to be risk factors for orofacial clefts. Furthermore, alcohol intake during pregnancy is a known teratogen, making the adjustment of these confounding risk factors even more critical (Carreras-Torres et al., 2018; Taylor et al., 2018, 2019).

Our meta-analysis suggests that maternal smoking may have a moderate role in CL/P etiology with pooled OR of 1.42 (95% CI: 1.27, 1.59). The PAF estimates the proportion of the disease that would be reduced by eliminating exposure to a given risk factor, assuming the risk factor is causal. The pooled PAF of 4% (95% CI: 3%, 5%) in this review is similar to the previously reported range of 4%-6% from three individual studies (Honein et al., 2007, 2014; Raut et al., 2019). The indication here is that should maternal smoking be eliminated, 4% of CL/P would not occur. Raut et al. (2019) reported maternal smoking to have the largest PAF for CL/P among 11 modifiable risk factors including maternal age, alcohol consumption, folic acid supplementation, obesity, maternal education, diabetes, and fever. The average-adjusted PAF, taking into account the combination of modifiable risk factors and additional nonmodifiable factors (such as sex and race) acting synergistically, was 50% for CL  ±  P and 43% for CP (Raut et al., 2019).

Evidence of a dose–response relationship can add support to a causal relationship. The analysis of dose effect in CL/P demonstrated the highest dose of smoking (>20 cigarettes per day) to have the strongest positive effect on risk of cleft, but the intermediate smoking dose (11-20 cigarettes per day) had a similar effect to the lowest dose (1-10 cigarettes per day). This may represent a threshold effect of more than 20 cigarettes needing to be smoked a day before a difference is noted in CL/P etiology. Alternatively, the greater effect in the highest smoking dose may reflect the propensity for risk-taking behaviors associated with additional confounding by substance abuse (such as alcohol), which may not have been adequately adjusted for. The dose of cigarettes per day was self-reported in all included studies, which introduces the potential for recall or reporting bias, and therefore reduces the validity of the measures. In addition, the effect of the highest smoking dose on CL/P etiology should be interpreted with caution as the number of cases within the individual studies were less than for low and medium smoking doses; therefore, the effect estimates were less precise.

Historically, CL/P has been subdivided in to CL  ±  P and CP, reflecting different embryological origins from the primary palate and secondary palate, respectively (Dixon et al., 2011). Studies included in this review reported individual outcomes for CL  ±  P and CP and the respective pooled ORs demonstrated a moderately positive association with maternal smoking, similar to that of OFC. The pooled OR for CP (OR  =  1.49) was greater than for CL  ±  P (OR  =  1.31) and this is an inverse of the relationship reported in two previous meta-analyses (Little et al., 2004; Xuan et al., 2016). The pooled OR for CP reported in this review should be interpreted with caution as it was influenced by the outlying result of a single study (Ács et al., 2020), with a heterogeneity between studies present. The only study with a good quality rating included in the CP meta-analysis (Raut et al., 2019), reported a more modest measure of effect; therefore, the pooled OR following exclusion of the outlying study (OR  =  1.25) may be a more accurate representation of the effect of smoking on CP etiology.

### Strengths and Limitations

Strengths of this review include a comprehensive search strategy with concerted efforts made to include all languages and a wide variety of study designs. Thorough assessment of study quality facilitated the inclusion of studies into the meta-analysis only if they met predefined threshold criteria.

The main limitation of interpreting the results from the meta-analysis relate to the inherent flaws of the standard analytical cohort and case–control approaches and their associated potential for bias. Studies were included in the meta-analysis if they had adjusted for a minimum set of confounders (maternal age and maternal alcohol consumption), which means that there was scope for additional important confounding factors to be unaccounted for. Even when adjustment for all relevant confounding factors is performed, bias may be present due to inaccurate measurement of confounding factors, misclassifications of exposure and differential missing data (Lawlor et al., 2016). The small sample sizes of some studies included in the meta-analysis meant their effect estimates were imprecise. A dose–response relationship could not be tested in CL  ±  P and CP outcomes due to differences in smoking dose categorization reported in the included studies. Restriction of the search to published studies could have introduced publication bias, despite the evidence for publication bias being weak. This review focused upon active cigarette smoking in females and while the association of both passive and paternal smoking on CL/P has been reported, there has been less scientific focus in these areas when compared to active maternal smoking (Savitz et al., 1991; Krapels et al., 2008; Figueiredo et al., 2015; Hao et al., 2015; Sabbagh et al., 2015).

### Interpretation

Our understanding of the causal role of maternal smoking in CL/P is limited because of biases affecting traditional observational methods and the impracticalities of performing randomized controlled trials in this setting. If our reported moderate association is an accurate reflection of the role that maternal smoking plays then we would predict that the elimination of this risk factor would result in the reduction of 8000 less cases per year worldwide as it is estimated that 200 000 children are born with CL/P per year (Mossey et al., 2009; The Central Intelligence Agency, 2021). This estimation is based on a 14% prevalence of maternal smoking in case mothers, originating from high-income country publications, whereas the World Health Organisation estimates 17% of the global population use tobacco products, mostly from low and middle-income countries (World Health Organisation, 2021).

The potential for maternal smoking to play a moderate role in CL/P etiology fits within our current understanding about the cause of CL/P being complex, multifactorial and involving both environmental and genetic factors (Dixon et al., 2011). Gene–environment interactions between smoking and CL/P have been the focus of a number of studies over the last two decades and these have improved our understanding of the pathogenesis of CL/P (Vieira, 2008; Krapels et al., 2008; Beaty et al., 2016; Garland et al., 2020). If smoking only accounts for 4% of the PAF, the environmental and genetic factors accounting for the remaining 96%, and the interplay between them, remains to be defined.

### Recommendations/Implications for Practice/Policy/Further Research

This review seeks to address an important public health question regarding the role of maternal smoking in CL/P etiology. Tobacco use is still common worldwide in pregnancy and is the focus of campaigns by the World Health Organization to reduce adverse health effects on woman and infants (World Health Organisation, 2013). The neonatal health risk associated with maternal smoking were highlighted to the public in 2014 by the U.S Surgeon General's Report, with smoking reported to increase the risk of CL/P by 30%-50% (United States Department of Health and Human Services, 2014). Focus group research has highlighted the difficulties of changing smoking behaviors in pregnant women but suggests educational information with pictorial representation of babies risk may be an effective motivational method (Levis et al., 2014).

The methodologies used by the 45 eligible studies were all conventional observational design (cohort or case–control designs). To strengthen our understanding of the causal role of maternal smoking in CL/P, this review highlights the need for high-quality studies using a variety of methodological approaches with different directions of bias (Pearce et al., 2019). An instrumental variable model using genetic variants as proxies for smoking has been used in the past to assess the effect of maternal smoking on CL/P risk and reported a substantially stronger positive effect than traditional analytic studies, but the genetic variants used were not strongly associated with smoking and the sample size was small (Wehby et al., 2011). More powerful studies, using multiple novel epidemiological designs that can overcome some of the limitations of traditional methods are required and have been used as part of a triangulated approach to further the understanding of the causal role of cigarette smoking for other health outcomes (Gage et al., 2020).

## Supplemental Material

sj-doc-1-cpc-10.1177_10556656211040015 - Supplemental material for Maternal Cigarette Smoking and Cleft Lip and Palate: A Systematic Review and Meta-Analysis Click here for additional data file. Supplemental material, sj-doc-1-cpc-10.1177_10556656211040015 for Maternal Cigarette Smoking and Cleft Lip and Palate: A Systematic Review and Meta-Analysis by Matthew Fell, Kyle Dack, Shaheel Chummun, Jonathan Sandy, Yvonne Wren and Sarah Lewis in The Cleft Palate-Craniofacial Journal

sj-docx-2-cpc-10.1177_10556656211040015 - Supplemental material for Maternal Cigarette Smoking and Cleft Lip and Palate: A Systematic Review and Meta-Analysis Click here for additional data file. Supplemental material, sj-docx-2-cpc-10.1177_10556656211040015 for Maternal Cigarette Smoking and Cleft Lip and Palate: A Systematic Review and Meta-Analysis by Matthew Fell, Kyle Dack, Shaheel Chummun, Jonathan Sandy, Yvonne Wren and Sarah Lewis in The Cleft Palate-Craniofacial Journal

sj-docx-3-cpc-10.1177_10556656211040015 - Supplemental material for Maternal Cigarette Smoking and Cleft Lip and Palate: A Systematic Review and Meta-Analysis Click here for additional data file. Supplemental material, sj-docx-3-cpc-10.1177_10556656211040015 for Maternal Cigarette Smoking and Cleft Lip and Palate: A Systematic Review and Meta-Analysis by Matthew Fell, Kyle Dack, Shaheel Chummun, Jonathan Sandy, Yvonne Wren and Sarah Lewis in The Cleft Palate-Craniofacial Journal

sj-png-4-cpc-10.1177_10556656211040015 - Supplemental material for Maternal Cigarette Smoking and Cleft Lip and Palate: A Systematic Review and Meta-Analysis Click here for additional data file. Supplemental material, sj-png-4-cpc-10.1177_10556656211040015 for Maternal Cigarette Smoking and Cleft Lip and Palate: A Systematic Review and Meta-Analysis by Matthew Fell, Kyle Dack, Shaheel Chummun, Jonathan Sandy, Yvonne Wren and Sarah Lewis in The Cleft Palate-Craniofacial Journal

sj-docx-5-cpc-10.1177_10556656211040015 - Supplemental material for Maternal Cigarette Smoking and Cleft Lip and Palate: A Systematic Review and Meta-Analysis Click here for additional data file. Supplemental material, sj-docx-5-cpc-10.1177_10556656211040015 for Maternal Cigarette Smoking and Cleft Lip and Palate: A Systematic Review and Meta-Analysis by Matthew Fell, Kyle Dack, Shaheel Chummun, Jonathan Sandy, Yvonne Wren and Sarah Lewis in The Cleft Palate-Craniofacial Journal

sj-png-6-cpc-10.1177_10556656211040015 - Supplemental material for Maternal Cigarette Smoking and Cleft Lip and Palate: A Systematic Review and Meta-Analysis Click here for additional data file. Supplemental material, sj-png-6-cpc-10.1177_10556656211040015 for Maternal Cigarette Smoking and Cleft Lip and Palate: A Systematic Review and Meta-Analysis by Matthew Fell, Kyle Dack, Shaheel Chummun, Jonathan Sandy, Yvonne Wren and Sarah Lewis in The Cleft Palate-Craniofacial Journal

sj-docx-7-cpc-10.1177_10556656211040015 - Supplemental material for Maternal Cigarette Smoking and Cleft Lip and Palate: A Systematic Review and Meta-Analysis Click here for additional data file. Supplemental material, sj-docx-7-cpc-10.1177_10556656211040015 for Maternal Cigarette Smoking and Cleft Lip and Palate: A Systematic Review and Meta-Analysis by Matthew Fell, Kyle Dack, Shaheel Chummun, Jonathan Sandy, Yvonne Wren and Sarah Lewis in The Cleft Palate-Craniofacial Journal

sj-png-8-cpc-10.1177_10556656211040015 - Supplemental material for Maternal Cigarette Smoking and Cleft Lip and Palate: A Systematic Review and Meta-Analysis Click here for additional data file. Supplemental material, sj-png-8-cpc-10.1177_10556656211040015 for Maternal Cigarette Smoking and Cleft Lip and Palate: A Systematic Review and Meta-Analysis by Matthew Fell, Kyle Dack, Shaheel Chummun, Jonathan Sandy, Yvonne Wren and Sarah Lewis in The Cleft Palate-Craniofacial Journal

sj-png-9-cpc-10.1177_10556656211040015 - Supplemental material for Maternal Cigarette Smoking and Cleft Lip and Palate: A Systematic Review and Meta-Analysis Click here for additional data file. Supplemental material, sj-png-9-cpc-10.1177_10556656211040015 for Maternal Cigarette Smoking and Cleft Lip and Palate: A Systematic Review and Meta-Analysis by Matthew Fell, Kyle Dack, Shaheel Chummun, Jonathan Sandy, Yvonne Wren and Sarah Lewis in The Cleft Palate-Craniofacial Journal

sj-jpg-10-cpc-10.1177_10556656211040015 - Supplemental material for Maternal Cigarette Smoking and Cleft Lip and Palate: A Systematic Review and Meta-Analysis Click here for additional data file. Supplemental material, sj-jpg-10-cpc-10.1177_10556656211040015 for Maternal Cigarette Smoking and Cleft Lip and Palate: A Systematic Review and Meta-Analysis by Matthew Fell, Kyle Dack, Shaheel Chummun, Jonathan Sandy, Yvonne Wren and Sarah Lewis in The Cleft Palate-Craniofacial Journal

sj-jpg-11-cpc-10.1177_10556656211040015 - Supplemental material for Maternal Cigarette Smoking and Cleft Lip and Palate: A Systematic Review and Meta-Analysis Click here for additional data file. Supplemental material, sj-jpg-11-cpc-10.1177_10556656211040015 for Maternal Cigarette Smoking and Cleft Lip and Palate: A Systematic Review and Meta-Analysis by Matthew Fell, Kyle Dack, Shaheel Chummun, Jonathan Sandy, Yvonne Wren and Sarah Lewis in The Cleft Palate-Craniofacial Journal

sj-jpg-12-cpc-10.1177_10556656211040015 - Supplemental material for Maternal Cigarette Smoking and Cleft Lip and Palate: A Systematic Review and Meta-Analysis Click here for additional data file. Supplemental material, sj-jpg-12-cpc-10.1177_10556656211040015 for Maternal Cigarette Smoking and Cleft Lip and Palate: A Systematic Review and Meta-Analysis by Matthew Fell, Kyle Dack, Shaheel Chummun, Jonathan Sandy, Yvonne Wren and Sarah Lewis in The Cleft Palate-Craniofacial Journal

sj-jpg-13-cpc-10.1177_10556656211040015 - Supplemental material for Maternal Cigarette Smoking and Cleft Lip and Palate: A Systematic Review and Meta-Analysis Click here for additional data file. Supplemental material, sj-jpg-13-cpc-10.1177_10556656211040015 for Maternal Cigarette Smoking and Cleft Lip and Palate: A Systematic Review and Meta-Analysis by Matthew Fell, Kyle Dack, Shaheel Chummun, Jonathan Sandy, Yvonne Wren and Sarah Lewis in The Cleft Palate-Craniofacial Journal
