# Supplementary material for: Maternal Cigarette Smoking and Cleft Lip and Palate: A Systematic Review and Meta-Analysis
Source: Cleft Palate Craniofac J. 2021 Sep 27;59(9):1185–200. doi: 10.1177/10556656211040015 (PMC9411693; doi:10.1177/10556656211040015)
Supplement: sj-docx-2-cpc-10.1177_10556656211040015 - Supplemental material for Maternal Cigarette Smoking and Cleft Lip and Palate: A Systematic Review and Meta-Analysis [file sj-docx-2-cpc-10.1177_10556656211040015.docx]

| **Supplementary Table 2: Criteria for including or excluding papers** | |
| --- | --- |
| **Include** | **Exclude** |
| **1. Publication:** Full-text papers published in a peer-reviewed journal. All languages included | **1. Publication:** Title, abstract or conference proceedings only or published in a non-peer reviewed journal (book, newspaper, or website) |
| **2. Study:** Primary data using analytical study designs (comparing exposed versus unexposed groups). These may include cohort, case-control, RCT, quasi experimental, Mendelian Randomization studies, natural experiment and family based negative control designs | **2. Study:** Descriptive studies (i.e. no comparison between exposed and unexposed groups). Approaches to exclude will be case studies, case series, cross-sectional studies, expert opinion, letters and editorials). Secondary data from reviews |
| **3. Population:** Pregnant women or women who have given birth to live born children in the general population | **3. Population:** Women who are not or have not been pregnant, partners/fathers and animal studies. |
| **4. Exposure:** Active cigarette smoking in women during pregnancy measured either by self-reporting or by proxy measurements | **4. Exposure:** Cigarette smoking before or after pregnancy but not explicitly during pregnancy. Intervention in study is not active cigarette smoking (i.e. cigar smoking, recreational drug smoking, vaping, passive/secondary smoking) |
| **5. Outcome:** children born with an orofacial cleft. This includes cleft lip, cleft palate, cleft lip and palate and submucous cleft palate.  **6. Measures of effect:** A calculation made to define the association between exposure and outcome | **5. Outcome:** Other offspring outcomes such as craniofacial abnormalities or developmental abnormalities  **6. Measures of effect:** No calculation made to define the association between exposure and outcome |
