# Supplementary material for: Maternal Cigarette Smoking and Cleft Lip and Palate: A Systematic Review and Meta-Analysis
Source: Cleft Palate Craniofac J. 2021 Sep 27;59(9):1185–200. doi: 10.1177/10556656211040015 (PMC9411693; doi:10.1177/10556656211040015)
Supplement: sj-docx-3-cpc-10.1177_10556656211040015 - Supplemental material for Maternal Cigarette Smoking and Cleft Lip and Palate: A Systematic Review and Meta-Analysis [file sj-docx-3-cpc-10.1177_10556656211040015.docx]

**Supplementary Table 3:** The Assignment of Stars for Study Quality using the Newcastle Ottawa Scale

| **Case Design** | **Domain** | **Criteria** | **Acceptable (Star Awarded)** | **Unacceptable (Star not awarded)** |
| --- | --- | --- | --- | --- |
| **Cohort** | Selection | Representativeness of the exposed cohort | Cohort represents or somewhat represents pregnant women in the general population | Selected group of pregnant women or no description of the derivation of the cohort |
|  |  | Selection of the non-exposed cohort | Pregnant women drawn from the same community as the exposed cohort | Pregnant women drawn from a different source or no description |
|  |  | Ascertainment of exposure | Structured interview or secure record (such as birth certificate) | Written self-report (i.e., survey) or no description |
|  |  | Outcome of interest was not present at start of study | Demonstrated | Not demonstrated |
|  | Comparability | Comparability of cohorts on the basis of the design or analysis | Study controls for maternal age and maternal alcohol consumption (for 1 star) and additionally for maternal folic acid supplementation and body mass index (for 2 stars) | Major confounding factors not controlled for |
|  | Outcome | Assessment of outcome | Orofacial cleft confirmed via record linkage or independent blind assessment | Orofacial cleft outcome confirmed by self-report or not stated |
|  |  | Was follow-up long enough for outcomes to occur | An adequate follow-up period was allocated after the birth of the baby to make a diagnosis of orofacial cleft | The cohort did not allow for follow-up on birth outcomes |
|  |  | Adequacy of follow up of cohorts | The follow up was >90% or a reasonable description for those lost to follow-up | The follow-up was <90% and no description for those lost to follow-up or not stated |
| **Case-Control** | Selection | Is the case definition adequate? | Diagnosis of cleft independently validated | Diagnosis of orofacial cleft made by record linkage alone or no description |
|  |  | Representativeness of the cases | All eligible cases of orofacial cleft over a defined period of time, in a defined catchment area or all cases from a treatment provider or a random sample taken | Case group selected is not consecutive or has potential for biased selection or not stated |
|  |  | Selection of Controls | Controls in the study selected from the same population as the cases | Controls derived from a hospitalised population or no description |
|  |  | Definition of Controls | Controls verified to have no history of orofacial cleft | No description |
|  | Comparability | Comparability of cases and controls on the basis of the design or analysis | Study controls for maternal age and maternal alcohol consumption (for 1 star) and additionally for maternal folic acid supplementation and body mass index (for 2 stars) | Major confounding factors not controlled for |
|  | Exposure | Ascertainment of exposure | Structured interview where blind to case/control status | Interview not blinded to case/control status or self-completed survey or no record |
|  |  | Ascertainment of exposure for cases and controls | Same method used for cases and controls | Different method used for cases and controls |
|  |  | Non-Response rate | Similar rate for cases and controls | Non-response rate appreciably different between cases and controls or not stated |
