# Supplementary figures and images for: Maternal Cigarette Smoking and Cleft Lip and Palate: A Systematic Review and Meta-Analysis
Source: Cleft Palate Craniofac J. 2021 Sep 27;59(9):1185–200. doi: 10.1177/10556656211040015 (PMC9411693; doi:10.1177/10556656211040015)

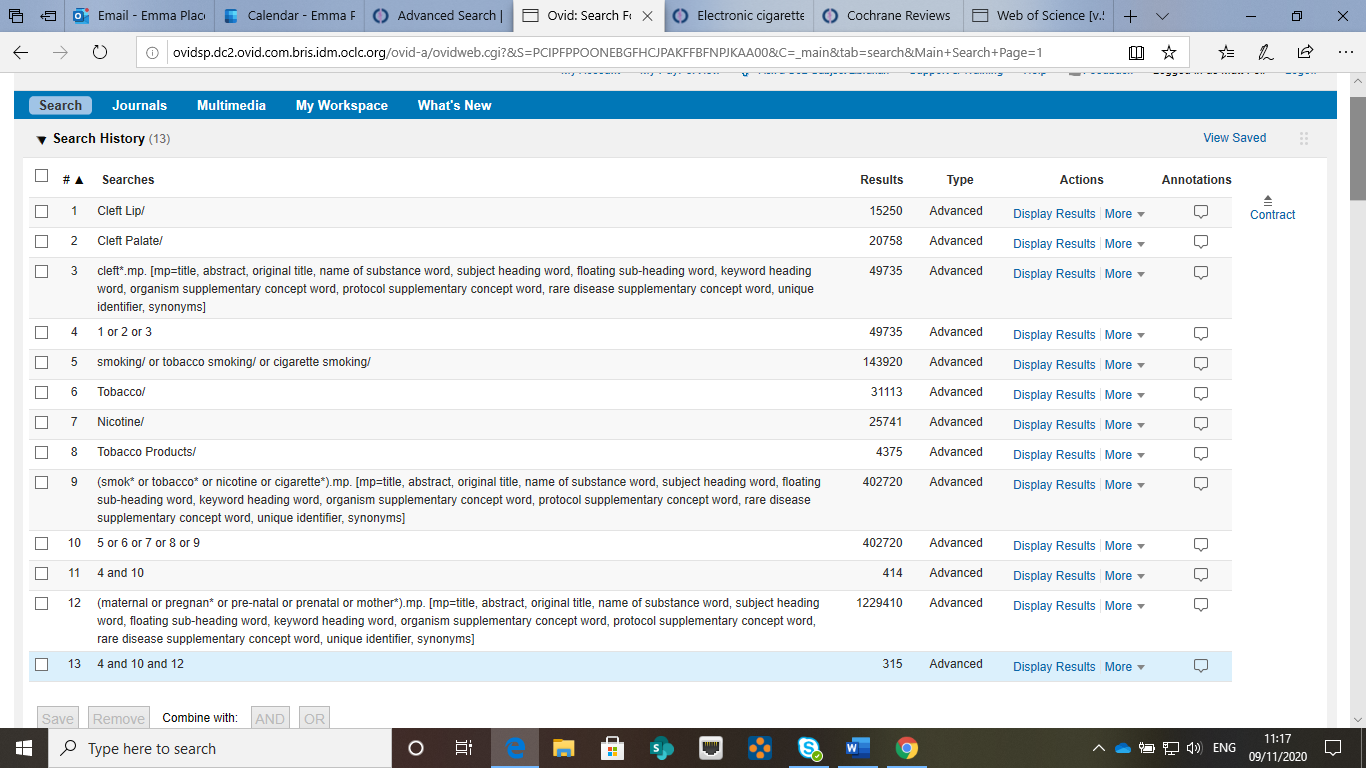

Supplement: sj-png-4-cpc-10.1177_10556656211040015 - Supplemental material for Maternal Cigarette Smoking and Cleft Lip and Palate: A Systematic Review and Meta-Analysis [file sj-png-4-cpc-10.1177_10556656211040015.png]

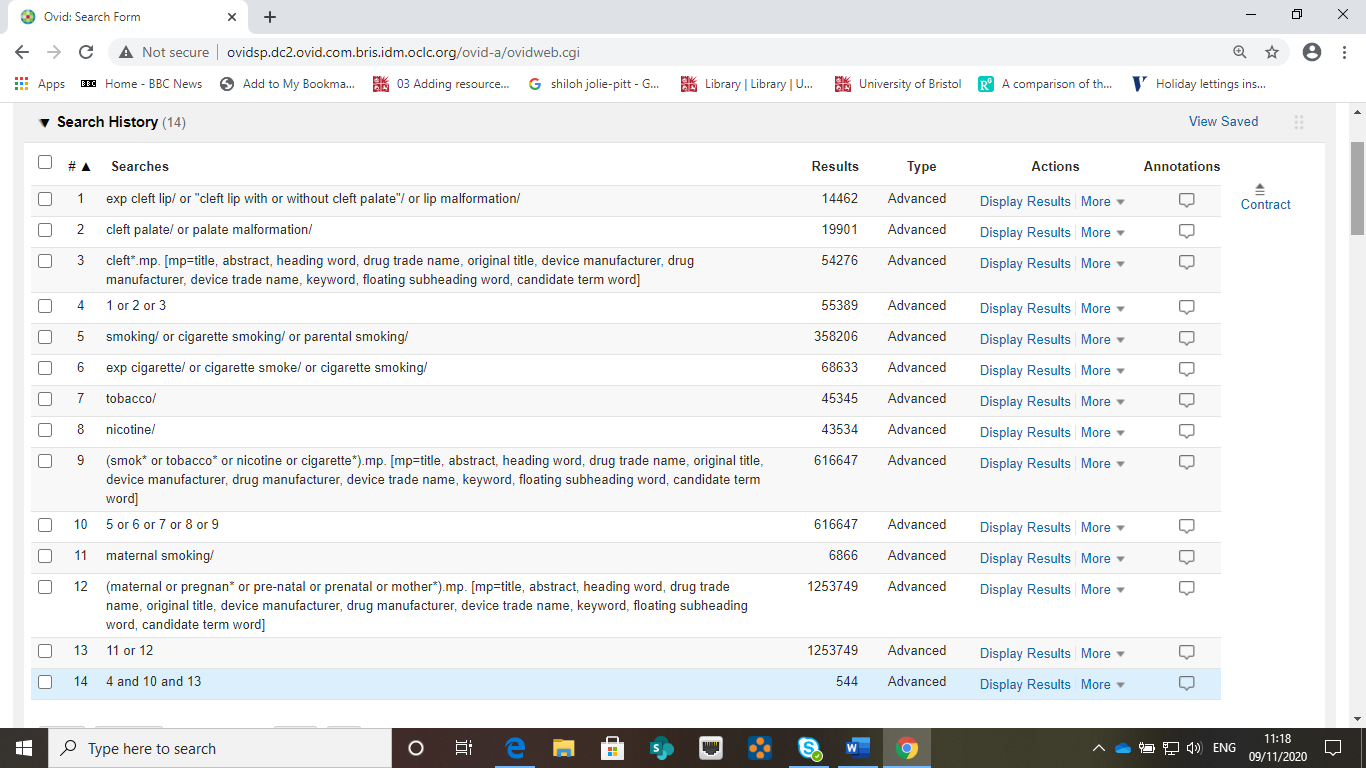

Supplement: sj-png-6-cpc-10.1177_10556656211040015 - Supplemental material for Maternal Cigarette Smoking and Cleft Lip and Palate: A Systematic Review and Meta-Analysis [file sj-png-6-cpc-10.1177_10556656211040015.png]

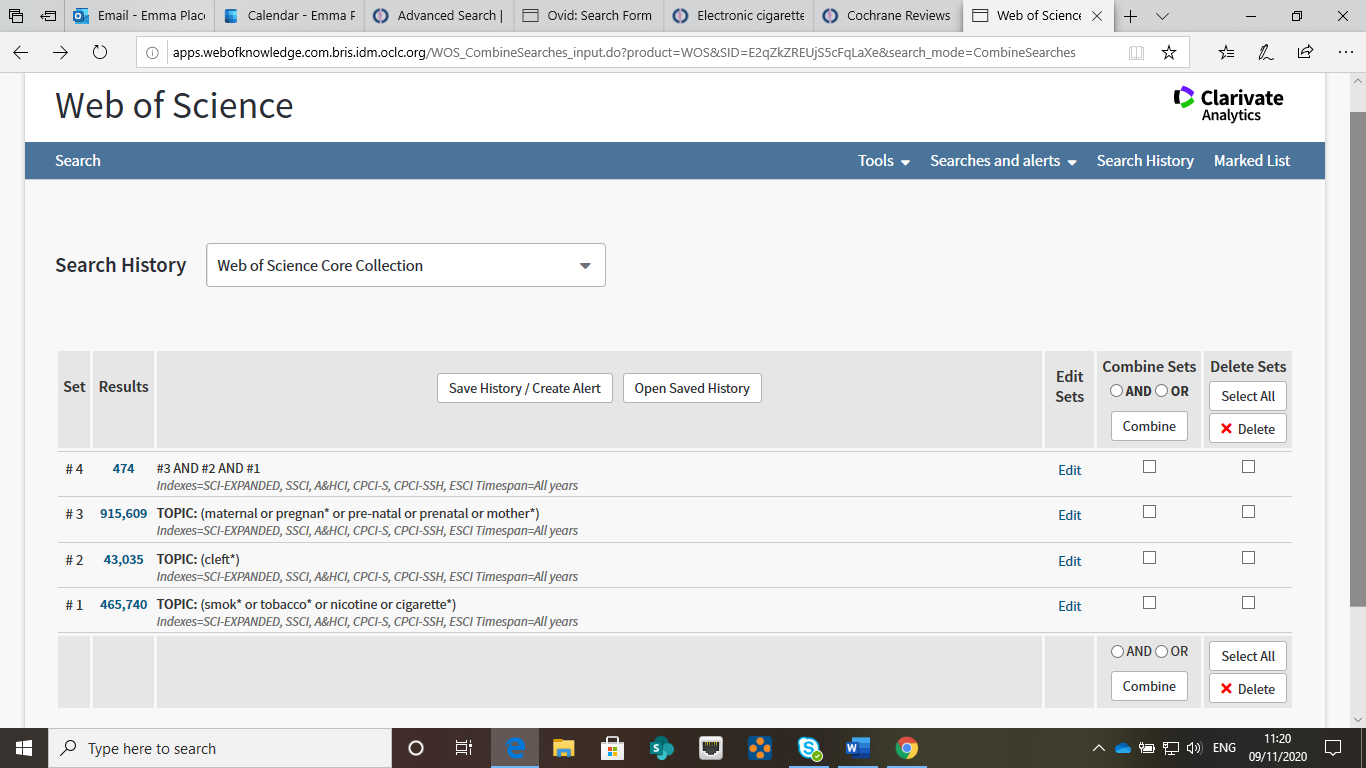

Supplement: sj-png-8-cpc-10.1177_10556656211040015 - Supplemental material for Maternal Cigarette Smoking and Cleft Lip and Palate: A Systematic Review and Meta-Analysis [file sj-png-8-cpc-10.1177_10556656211040015.png]

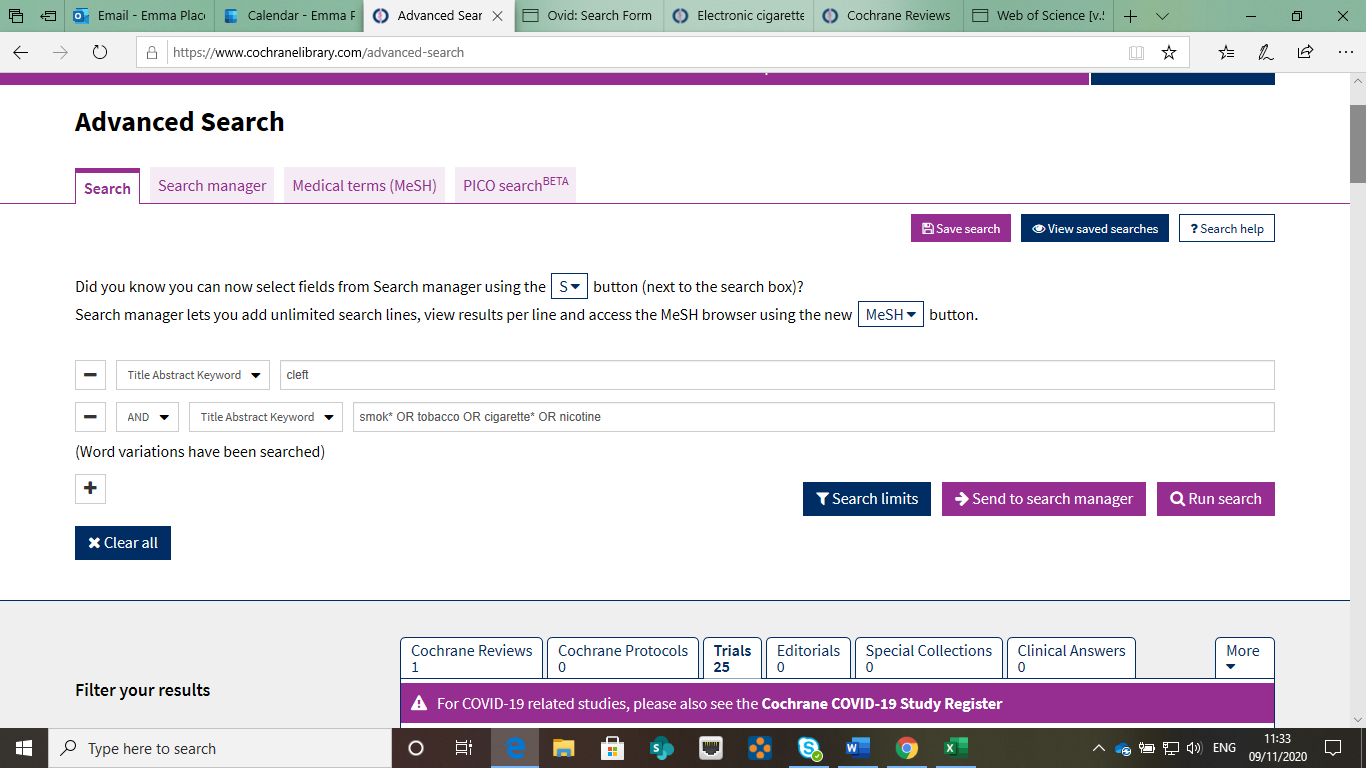

Supplement: sj-png-9-cpc-10.1177_10556656211040015 - Supplemental material for Maternal Cigarette Smoking and Cleft Lip and Palate: A Systematic Review and Meta-Analysis [file sj-png-9-cpc-10.1177_10556656211040015.png]

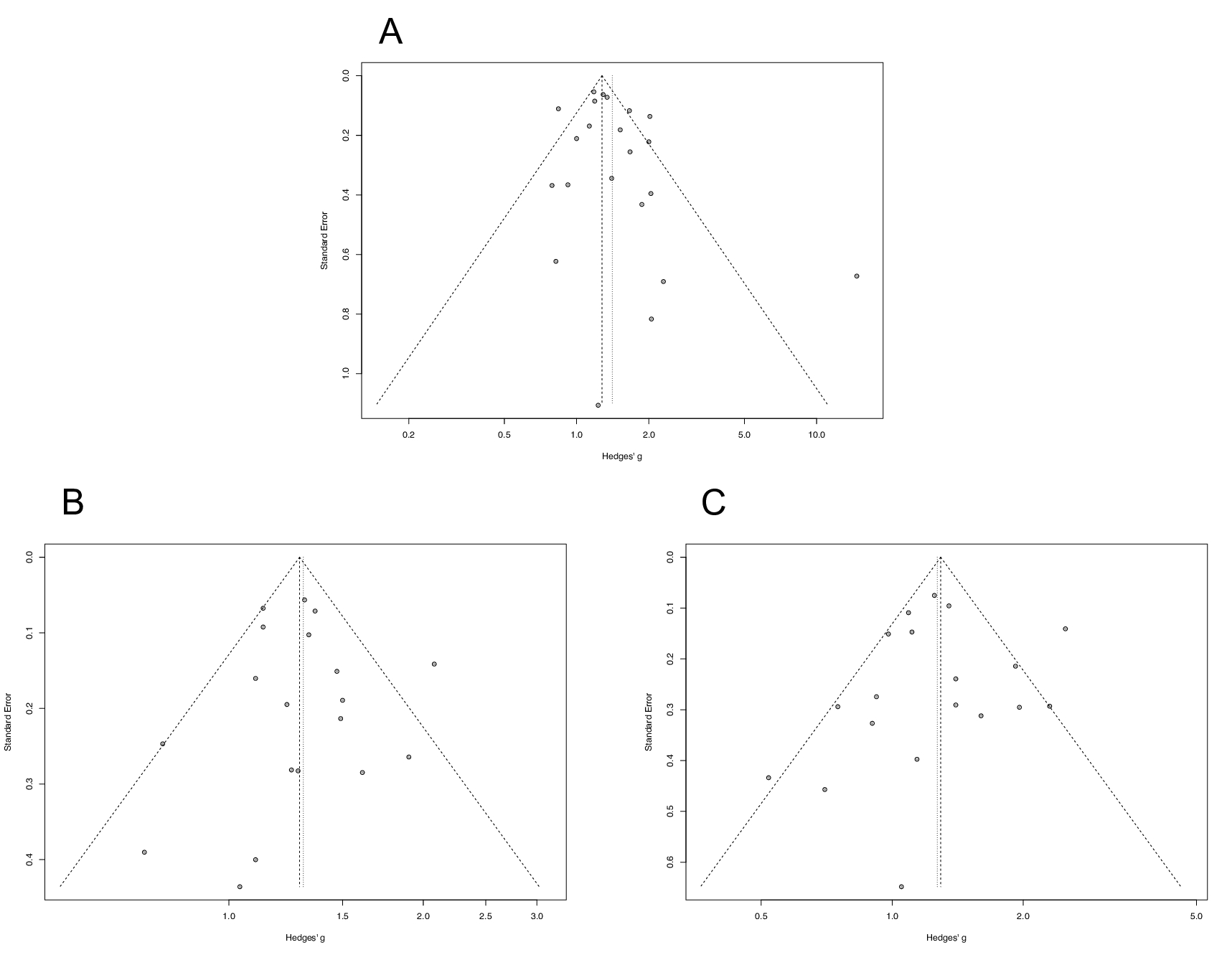

Supplement: sj-jpg-10-cpc-10.1177_10556656211040015 - Supplemental material for Maternal Cigarette Smoking and Cleft Lip and Palate: A Systematic Review and Meta-Analysis [file sj-jpg-10-cpc-10.1177_10556656211040015.jpg]

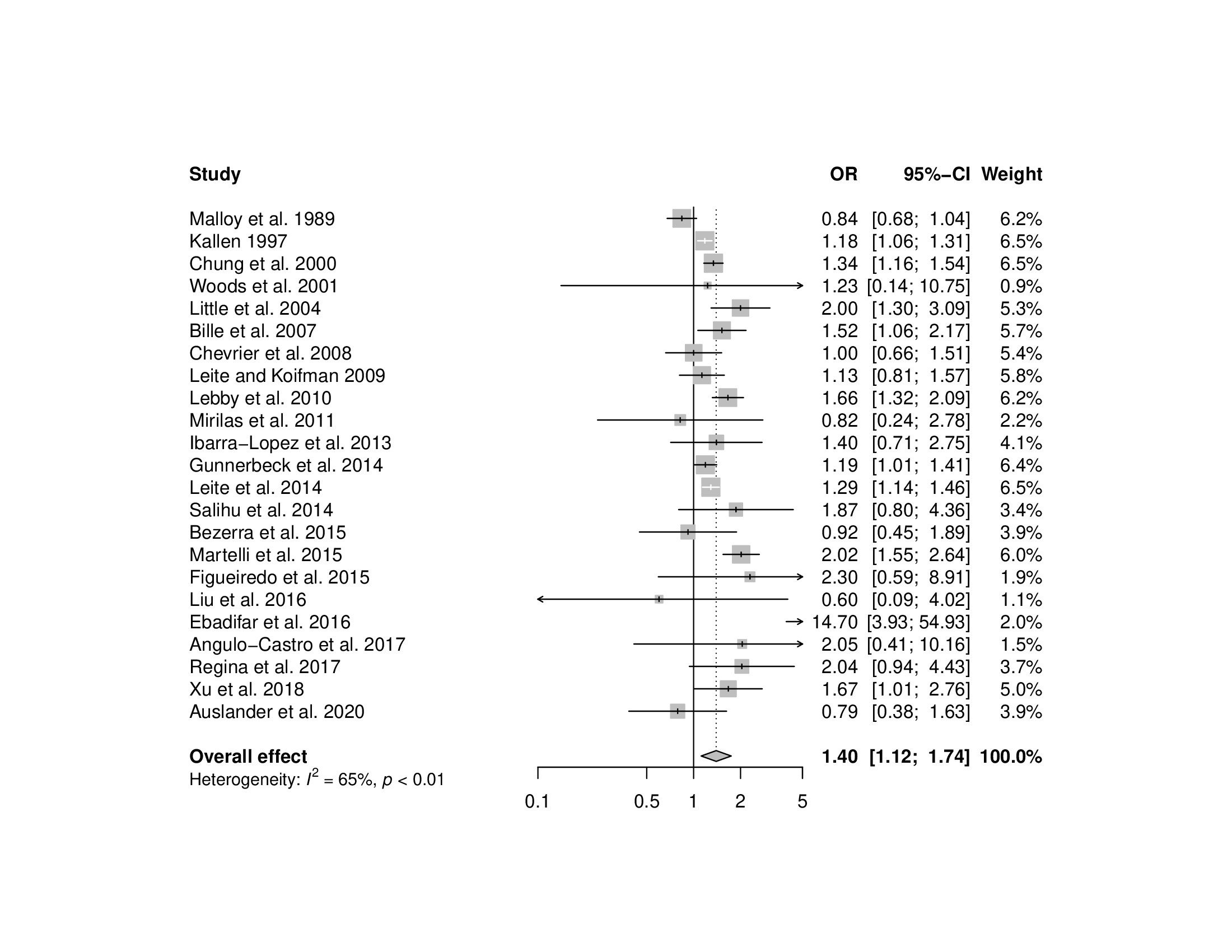

Supplement: sj-jpg-11-cpc-10.1177_10556656211040015 - Supplemental material for Maternal Cigarette Smoking and Cleft Lip and Palate: A Systematic Review and Meta-Analysis [file sj-jpg-11-cpc-10.1177_10556656211040015.jpg]

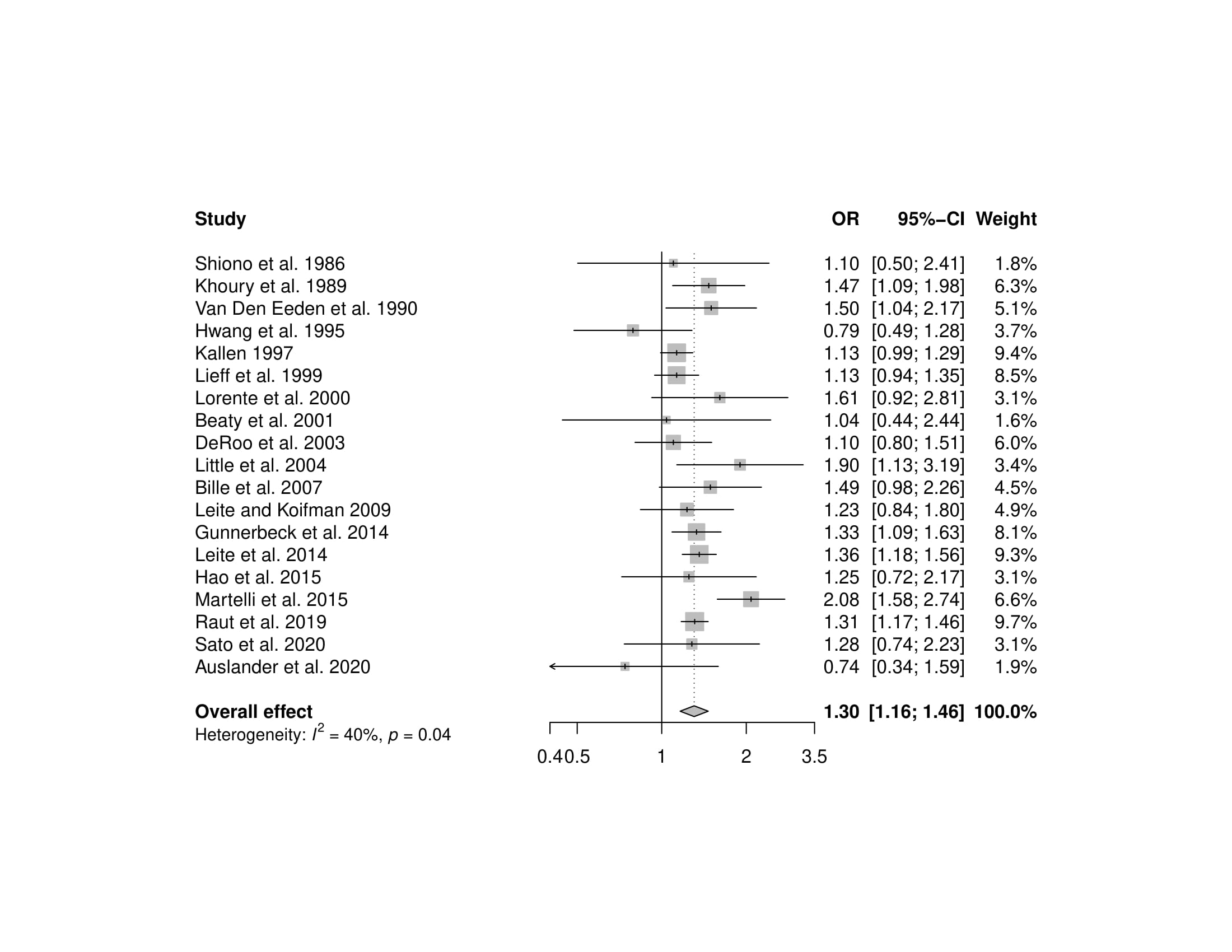

Supplement: sj-jpg-12-cpc-10.1177_10556656211040015 - Supplemental material for Maternal Cigarette Smoking and Cleft Lip and Palate: A Systematic Review and Meta-Analysis [file sj-jpg-12-cpc-10.1177_10556656211040015.jpg]

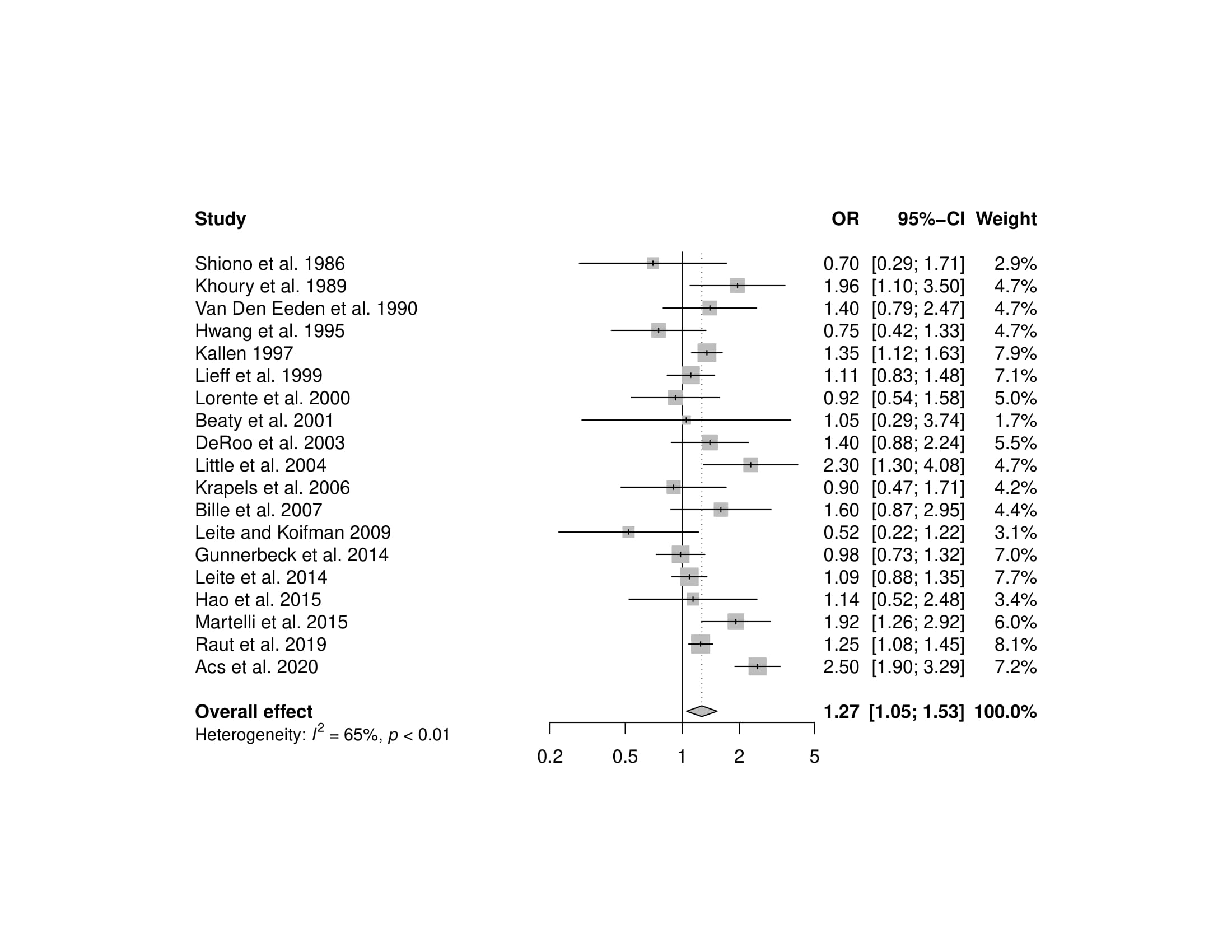

Supplement: sj-jpg-13-cpc-10.1177_10556656211040015 - Supplemental material for Maternal Cigarette Smoking and Cleft Lip and Palate: A Systematic Review and Meta-Analysis [file sj-jpg-13-cpc-10.1177_10556656211040015.jpg]
