# Supplementary material for: Maternal Cigarette Smoking and Cleft Lip and Palate: A Systematic Review and Meta-Analysis
Source: Cleft Palate Craniofac J. 2021 Sep 27;59(9):1185–200. doi: 10.1177/10556656211040015 (PMC9411693; doi:10.1177/10556656211040015)
Supplement: sj-docx-5-cpc-10.1177_10556656211040015 - Supplemental material for Maternal Cigarette Smoking and Cleft Lip and Palate: A Systematic Review and Meta-Analysis [file sj-docx-5-cpc-10.1177_10556656211040015.docx]

**Supplementary Table 4:** Articles Excluded at the Full Text Screening Stage and Reasons for Exclusion

| **Author** | **Year** |  | **Publication Title** | **Reason for exclusion** | **Explanation** |
| --- | --- | --- | --- | --- | --- |
| Saxen | 1974 |  | Cleft lip and palate in Finland: Parental histories, course of pregnancy and selected environmental factors | Study design | No comparison group |
| Evans et al. | 1979 |  | Maternal smoking habits and congenital malformations: A population study | Association | No measure of effect calculated between exposure and outcome |
| Ericson et al. | 1979 |  | Cigarette smoking as an etiologic factor in cleft lip and palate | Association | No measure of effect calculated between exposure and outcome |
| Christianson et al. | 1980 |  | The relationship between maternal smoking and the incidence of congenital anomalies | Outcome | Cleft not specified as an outcome studied |
| Hemminki et al | 1983 |  | Smoking and the occurrence of congenital malformations and spontaneous abortions: Multivariate analysis | Association | Incomplete measure of effect calculation |
| Niebyl et al. | 1985 |  | Lack of maternal metabolic, endocrine, and environmental influences in the etiology of cleft lip with or without cleft palate | Association | No measure of effect calculated between exposure and outcome |
| Khoury et al. | 1987 |  | Maternal cigarette smoking and oral clefts: A population-based study | Multiple report of study | Crossover with Hwang et al., 1995 |
| Werler et al. | 1990 |  | Maternal cigarette smoking during pregnancy in relation to oral clefts | Multiple report of study | Crossover with Lieff et al 1999 |
| Loffredo et al. | 1994 |  | Oral clefts - a case-control study | Exposure | No calculation for smoking as an exposure of interest |
| Munger et al. | 1996 |  | Maternal alcohol use and risk of orofacial cleft birth defects | Exposure | No calculation for smoking as an exposure of interest |
| Beaty et al | 1997 |  | Testing for Interaction between Maternal Smoking and TGFA Genotype among Oral Cleft Cases Born in Maryland 1992–1996 | Multiple report of study | Crossover with Beaty et al., 2001 |
| Lieff et al. | 1999 |  | Selection bias and the use of controls with malformations in case- control studies of birth defects | Multiple report of study | Crossover with Lieff et al, 1999 |
| Romitti et al. | 1999 |  | Candidate genes for non-syndromic cleft lip and palate and maternal cigarette smoking and alcohol consumption: Evaluation of genotype-environment interactions from a population-based case-control study of orofacial clefts | Multiple report of study | Crossover with Shi et al 2007 |
| Christensen et al. | 1999 |  | Oral clefts, transforming growth factor alpha gene variants, and maternal smoking: A population-based case-control study in Denmark, 1991- 1994 | Multiple report of study | Crossover with Shi et al 2007 |
| Kallen | 2000 |  | Multiple malformations and maternal smoking | Outcome | Cleft not an outcome |
| Van Rooij et al. | 2001 |  | Smoking, genetic polymorphisms in biotransformation enzymes, and non-syndromic oral clefting: A gene-environment interaction | Multiple report of study | Crossover with Krapels et al., 2006 |
| Yoon et al. | 2001 |  | The National Birth Defects Prevention Study | Association | No association calculated between exposure and outcome |
| Kallen | 2002 |  | Maternal smoking and congenital malformations | Association | Incomplete measure of effect calculation |
| Van Rooij et al. | 2002 |  | Orofacial clefts and spina bifida: N-acetyltransferase phenotype, maternal smoking, and medication use | Multiple report of study | Crossover with Van Rooij et al., 2001 which has a greater number of patients |
| Werler et al. | 2003 |  | Findings on potential teratogens from a case-control study in Western Australia | Outcome | Cleft not specified as an outcome studied |
| Lammer et al | 2004 |  | Maternal smoking and the risk of orofacial clefts: Susceptibility with NAT1 and NAT2 polymorphisms | Multiple report of study | Crossover with Shaw et al., 1996, which had the primary data |
| Bille et al. | 2005 |  | Changing lifestyles and oral clefts occurrence in Denmark | association | No association calculated between exposure and outcome |
| Rouget et al. | 2005 |  | Periconceptional folates and the prevention of orofacial clefts: Role of dietary intakes in France | Multiple report of study | Crossover with Chevrier et al., 2008 |
| Lammer et al. | 2005 |  | Maternal smoking, genetic variation of glutathione S-transferases, and risk for orofacial clefts | Multiple report of study | Crossover with Shaw et al., 1996, which had the primary data |
| Shaw et al. | 2005 |  | Endothelial nitric oxide synthase (NOS3) genetic variants, maternal smoking, vitamin use, and risk of human orofacial clefts | Multiple report of study | Crossover with Shaw et al., 1996, which had the primary data |
| Wilcox et al. | 2007 |  | Folic acid supplements and risk of facial clefts: National population based case-control study | Exposure | No calculation for smoking as an exposure of interest |
| Honein et al. | 2007 |  | Maternal smoking and environmental tobacco smoke exposure and the risk of orofacial clefts | Multiple report of study | Crossover with MacLehose et al., 2009 which is an updated dataset |
| Mossey et al. | 2007 |  | Prevention of orofacial clefts: Does pregnancy planning have a role? | Exposure | No calculation for smoking as an exposure of interest |
| Ramirez et al. | 2007 |  | Maternal smoking during early pregnancy, GSTP1 and EPHX1 variants, and risk of isolated orofacial clefts | Exposure | In this gene-association study, smoking was only analysed within genetic subgroups |
| Gebreab et al. | 2008 |  | Visualization and interpretation of birth defects data using linked micromap plots | Study design | Descriptive study |
| Shaw et al. | 2009 |  | Mid-Pregnancy Cotinine and Risks of Orofacial Clefts and Neural Tube Defects | Exposure | No calculation for active smoking estimation |
| MacLehose et al. | 2009 |  | Bayesian methods for correcting misclassification: An example from birth defects epidemiology | Multiple report of study | Crossover with Raut et al., 2019 |
| Marshall et al. | 2010 |  | Oral cleft defects and maternal exposure to ambient air pollutants in New Jersey | Exposure | Active smoking was not the exposure |
| Munger et al. | 2011 |  | Oral clefts and maternal biomarkers of folate-dependent one-carbon metabolism in Utah | Exposure | No calculation for smoking as an exposure of interest |
| Zhang et al. | 2011 |  | Cigarette smoke exposure before pregnancy and the associated risk of having a child with orofacial clefts in china: A case-control study | Publication type | Abstract only |
| Bahado-Singh et al. | 2011 |  | Male gender significantly increases risk of oxidative stress related congenital anomalies in the non-diabetic population | Exposure | Maternal smoking not assessed as an independent exposure |
| Wehby et al. | 2011 |  | Genes as instruments for studying risk behavior effects: An application to maternal smoking and orofacial clefts | Multiple report of study | Primary data from Lie et al., 2008 |
| Zandi et al. | 2011 |  | An epidemiologic study of orofacial clefts in Hamedan City, Iran: A 15-year study | Exposure | No smokers in the control group |
| Reiter et al. | 2012 |  | Genetic and environmental risk factors for submucous cleft palate | Study design | Secondary data used for the control group |
| Taghavi et al. | 2012 |  | Orofacial clefts and risk factors in Tehran, Iran: A case control stud | Exposure | Population described as ex-smokers rather than current smokers |
| Buyu et al. | 2012 |  | Orofacial clefts at Bugando Medical Centre: Associated factors and postsurgical complications | Association | No association calculated between exposure and outcome |
| Jurek et al. | 2013 |  | Adjusting for outcome misclassification: The importance of accounting for case-control sampling and other forms of outcome-related selection | Study design | Secondary data used |
| Jurek et al. | 2013 |  | Adjusting for multiple-misclassified variables in a study using birth certificates | Study design | Secondary data used |
| Campos et al. | 2016 |  | Environmental factors related to the occurrence of oral clefts in a Brazilian subpopulation | Study design | Descriptive study |
| Kummet et al. | 2016 |  | Passive Smoke Exposure as a Risk Factor for Oral Clefts-A Large International Population-Based Study | Study design | Secondary data used |
| Sabbagh et al. | 2016 |  | Environmental risk factors in the aetiology of non-syndromic orofacial clefts in the western region of Saudi Arabia | Population | Paternal smoking |
| Wehby et al. | 2017 |  | Interaction between smoking and body mass index and risk of oral clefts | Study design | Secondary data used |
| Lili et al. | 2017 |  | Association between non-syndromic cleft lip with or without cleft palate and environmental factors in Ningxia | Population | Paternal smoking |
| Gao et al. | 2017 |  | Do smoking bans improve infant health? Evidence from U.S. Births: 1995-2009 | Association | No association calculated between exposure and outcome |
| Silva et al. | 2018 |  | Risk factors and comorbidities in Brazilian patients with orofacial clefts | Study design | Descriptive study |
| Crossan et al. | 2018 |  | Is there an association between maternal smoking and oral clefts? | Publication type | Review |
| Bui et al. | 2018 |  | Association Between Cleft Lip and/or Cleft Palate and Family History of Cancer: A Case-Control Study | Population | No defined maternal smoking group |
| Bui et al. | 2018 |  | Maternal Tobacco Exposure and Development of Orofacial Clefts in the Child | Population | Paternal smoking |
| Acs et al. | 2019 |  | First data from the new, unified database of the Hungarian case-control surveillance of congenital abnormalities | Association | No association calculated between exposure and outcome |
| Dastgiri et al. | 2019 |  | Estimation of the preventable proportion of congenital anomalies by selected risk factors in mothers: A case study in Iran | Population | No definition of maternal smoking group |
| Yu et al. | 2019 |  | Birth anomalies in monozygotic and dizygotic twins: Results from the California twin registry | Exposure | Smoking was a cofactor and not an independently studied exposure |
| Chowchuen et al. | 2020 |  | Birth Prevalence and Risk Factors Associated With CL/P in Thailand | Exposure | No association calculation for active smoking |
| Hong et al. | 2020 |  | Environmental Risk Factors for Non-syndromic Cleft Lip and/or Cleft Palate in Xinjiang Province, China: A Multi-ethnic Study | Population | Paternal smoking |
| Kruse et al. | 2020 |  | Impact of Maternal Smoking on Non-syndromic Clefts: Sex-Specific Associations With Side and Laterality | Study design | Descriptive study |
| Heinke et al. | 2020 |  | Quantification of selection bias in studies of risk factors for birth defects among livebirths | Multiple report of study | Crossover with Raut et al., 2019 |
