# Supplementary material for: Maternal Cigarette Smoking and Cleft Lip and Palate: A Systematic Review and Meta-Analysis
Source: Cleft Palate Craniofac J. 2021 Sep 27;59(9):1185–200. doi: 10.1177/10556656211040015 (PMC9411693; doi:10.1177/10556656211040015)
Supplement: sj-docx-7-cpc-10.1177_10556656211040015 - Supplemental material for Maternal Cigarette Smoking and Cleft Lip and Palate: A Systematic Review and Meta-Analysis [file sj-docx-7-cpc-10.1177_10556656211040015.docx]

**Supplementary Table 5:** The adjustment for four confounding factors was assessed in the comparability domain of the Newcastle Ottawa Scale; these were maternal age, maternal alcohol consumption, folic acid supplementation and maternal obesity. The diamond markers indicate adjustment for these factors in the individual studies included in this review. Additional confounding factors adjusted for are listed.

| **Author** | **Year** | **Maternal age** | **Alcohol** | **Folic Acid** | **Obesity** | **Additional confounding factors included** |
| --- | --- | --- | --- | --- | --- | --- |
| Shiono et al. | 1986 | ⯁ | ⯁ |  |  | Race |
| Khoury et al. | 1989 | ⯁ | ⯁ |  |  | Race, maternal education, planned pregnancy,  oral contraceptive use, tranquilizer use, spermicide use |
| Malloy et al. | 1989 | ⯁ |  |  |  | Marital status, education, parity |
| Van Den Eeden et al. | 1990 | ⯁ | ⯁ |  |  | Race, paternal age, marital status, parity, prior fetal loss, month prenatal care began |
| McDonald et al. | 1992 | ⯁ | ⯁ |  |  | Race, caffeine, educational level |
| Hwang et al. | 1995 | ⯁ |  |  |  | Parity |
| Shaw et al. | 1996 | ⯁ | ⯁ | ⯁ |  | Race, Maternal education, gravity, diabetes |
| Kallen | 1997 | ⯁ |  |  |  | Parity |
| Lieff et al. | 1999 |  |  |  |  | Family history of cleft, Infant sex, education level, maternal seizures,  ectopic pregnancy, miscarriage, therapeutic abortion,  still birth |
| Lorente et al. | 2000 | ⯁ | ⯁ |  |  | Socioeconomic status, area of residence |
| Chung et al. | 2000 | ⯁ | ⯁ |  |  | Race, maternal education, diabetes, diabetes |
| Beaty et al. | 2001 | ⯁ | ⯁ | ⯁ |  | Race, Maternal education |
| Woods et al. | 2001 | ⯁ |  |  |  | Race, Diabetes |
| Wyszynski and Wu | 2002 | ⯁ | ⯁ |  |  | Nil |
| DeRoo et al. | 2003 | ⯁ |  |  |  | Race, Infant sex, marital status |
| Little et al. | 2004 |  | ⯁ | ⯁ |  | Race, Infant sex, season of birth, maternal education |
| Meyer et al. | 2004 | ⯁ |  |  |  | Parity, diabetes race, |
| Krapels et al. | 2006 |  | ⯁ | ⯁ |  | Maternal medications |
| Shi et al. | 2007 |  | ⯁ | ⯁ |  | Nil |
| Shi et al. | 2007 | ⯁ | ⯁ |  |  | Family history of cleft, maternal education, gravidity |
| Bille et al. | 2007 | ⯁ | ⯁ | ⯁ |  | Social class |
| Grewal et al. | 2008 | ⯁ |  | ⯁ | ⯁ | Race, maternal education, gravidity and employment |
| Li et al. | 2008 |  | ⯁ | ⯁ |  | Maternal education, work status, fathers income,  calendar year of birth. |
| Chevrier et al. | 2008 |  | ⯁ | ⯁ |  | Child sex, geographical origin and maternal education |
| Leite and Koifman | 2009 | ⯁ | ⯁ |  |  | Maternal education |
| Lebby et al. | 2010 |  | ⯁ |  |  | Race, maternal education, hypertension, diabetes,  behavioural factors |
| Mirilas et al. | 2011 |  |  |  |  | NIL |
| Zhang et al. | 2011 | ⯁ | ⯁ | ⯁ |  | Maternal education and baby sex |
| Wehby et al. | 2011 |  | ⯁ | ⯁ | ⯁ | Planned pregnancy |
| Ibarra-Lopez et al. | 2013 |  |  | ⯁ |  | Maternal Education, socioeconomic status |
| Gunnerbeck et al. | 2014 | ⯁ |  |  |  | Parity, maternal education |
| Leite et al. | 2014 | ⯁ |  |  | ⯁ | Calendar time, marital status |
| Salihu et al. | 2014 | ⯁ |  |  |  | Family history of cleft, maternal medications |
| Bezerra et al. | 2015 |  | ⯁ |  |  | Family history of cleft |
| Hao et al. | 2015 | ⯁ | ⯁ |  | ⯁ | Maternal education, parity, use of medication |
| Martelli et al. | 2015 |  |  |  |  | Infant gender |
| Figueiredo | 2015 |  | ⯁ |  |  | Race, marital status, maternal education, parity, maternal drug use |
| Ebadifar et al. | 2016 | ⯁ |  |  |  | Maternal education, consanguinity, infant sex |
| Liu et al. | 2016 |  |  |  |  | Family history of cleft, occupation, maternal education, infant sex, multiple births |
| Angulo-Castro et al. | 2017 |  | ⯁ | ⯁ |  | Gestational age, birth weight, multivitamins,  maternal drug use, history of STIs, marital status,  socioeconomic status, maternal education, nutritional status |
| Xu et al. | 2018 | ⯁ | ⯁ | ⯁ |  | Family history of cleft, maternal past medical history, vitamin intake,  medications, fever, infection, radiation contact |
| Raut et al. | 2019 | ⯁ | ⯁ | ⯁ | ⯁ | Family history of cleft, race, maternal education, diabetes, previous pregnancies,  fever, infant sex |
| Sato et al. | 2020 | ⯁ | ⯁ | ⯁ | ⯁ | Baby sex, maternal education, socioeconomic |
| Acs et al. | 2020 | ⯁ | ⯁ |  |  | Geography of living, parity, maternal disease, drug intake |
| Regina et al | 2020 |  | ⯁ | ⯁ | ⯁ | Race, child gender, maternal education, gravity,  diabetes, hypertension, use of medications, radiation |
| Ausländer et al. | 2020 | ⯁ | ⯁ | ⯁ | ⯁ | Family history of cleft, maternal medical and medication history,  gravidity and maternal education |
